# Microbial composition, assembly, and functional characteristics of generalized and specialized subcommunities under flooded paddy fields: long-term pesticide versus non-pesticide models

**DOI:** 10.3389/fmicb.2025.1636555

**Published:** 2025-07-10

**Authors:** Jing-Wen Wang, Cai-Ting Han, Dong-Ming Wu, Wei Zhou, Miao Gao, Yu-Shuang Li

**Affiliations:** ^1^State Key Laboratory of Efficient Utilization of Arid and Semi-Arid Arable Land in Northern China, Institute of Agricultural Resources and Regional Planning, Chinese Academy of Agricultural Sciences, Beijing, China; ^2^Key Lab of Plant Nutrition and Fertilizer, Key Laboratory of Microbial Resources Collection and Preservation, Ministry of Agriculture and Rural Affairs, Beijing, China; ^3^Key Laboratory of Regional Environment and Eco-Remediation of Ministry of Education, College of Environment, Shenyang University, Shenyang, China; ^4^Hainan Key Laboratory of Tropical Eco-Circuling Agriculture, Environment and Plant Protection Institute, Chinese Academy of Tropical Agricultural Sciences, Haikou, China

**Keywords:** pesticide pollution, microbial community generalists and specialist, soil health, microbial network, microbial function

## Abstract

**Background:**

The extensive application of pesticides in agricultural practices is known to affect microbial health and thus eco-multifunctionality in soil. However, previous studies have mainly focused on these effects on whole microbial community in dryland, without considering that in paddy fields under flooded condition, especially lacking the finer insights into subcommunities related to niche fitness.

**Methods:**

To address this issue, the paddy fields, managed with (HP) and without pesticide application (HH) over 8 years, were selected. Then, the occurrence characteristics, function and assembly of generalized and specialized subcommunities classified by niche fitness were investigated.

**Results:**

The findings revealed that compared to HP model, the microbiota under HH model displayed higher bacterial diversity in both specialists and generalists, as well as greater fungal diversity in the generalists. However, pesticide residues in HP treatment increased copiotrophic microorganisms (e.g., *Gemmatimonadota*) in paddy soil, whereas oligotrophic microorganisms (e.g., *Proteobacteria* and *Acidobacteriota*) were significantly reduced. This indicated a special response of microflora to pesticide application under flooded condition, challenging the traditional views. Despite these changes, HP treatment also exhibited a substantial rise in pathogenic *Fusarium*, implying a higher diseases risk for rice. Neutral modeling revealed that both bacterial and fungal communities in HP were primarily driven by deterministic processes. Especially for specialists, it had a homogenization selection and diffusional limitation during community succession, because it was characterized by constrained adaptability, narrow ecological niches and high resource specificity. Regarding microbial networks, HP treatment resulted in lower node degree and closeness centrality compared to HH, leading to a decline in the overall functional capacity of the microbial community. FAPROTAX functional predictions further observed a significant reduction in the genes associated with nitrogen cycle and cellulolysis, while the human disease-related genes were increased in HP treatment. Collectively, these findings reveal that pesticide application in paddy fields significantly impacts microbial community structure and function, with specialized subcommunities being particularly vulnerable. These findings broaden our understanding into assembly principles of specialists and generalists under pesticide application in flooded paddy, which will contribute to the sustainable management for rice cultivation.

## 1 Introduction

To meet the increasing demand for food, substantial quantities of pesticides are applied to agricultural soils. According to the report, the global use of agricultural pesticides reached 3.7 million tons in 2022, with an average use of 2.38 kg/ha, an increase of 3% over 2021 (Tang et al., 2021). Due to the non-target toxicity of many pesticides, a large number of pesticides will accumulate in the ecosystem through bioenrichment and biomagnification after entering the environment. This will not only destroy the ecological balance of soil and water, but also affect human health through the food chain, leading to chronic poisoning, endocrine disorders, nervous system damage, and even cancer, teratogenesis and other problems (Cech et al., 2023; Maggi et al., 2021). Ultimately, these hazards will threaten the stability of the entire ecosystem and the living environment of human beings. In particular, microbes, as key components of soil ecosystems, are of particular concern (Mu et al., 2023). It is not only the core of soil nutrient cycling, driving the transformation and cycling process of important elements such as nitrogen and phosphorus in the soil, but also as an assistant to plant growth, promoting plant growth through nitrogen fixation, phosphorus solution, synthesis of plant hormones and other ways (Song et al., 2018). However, due to their low abundance, high sensitivity and rapid response to environmental changes, microorganisms are more sensitive to pesticide hazards. Studies have shown that, pesticides exert direct toxicity on target organisms through mechanisms such as enzyme inhibition, receptor binding, metabolic interference, and oxidative stress. Meanwhile, they indirectly affect soil ecological functions by inducing changes in microbial community structure and altering the abundance of functional genes. Lv et al. (2025) found that exposure to thiazole pesticides would weaken the proportion of rare microbial groups in the keystone nodes of the microbial molecular ecological network, posing a potential risk to the stability of agricultural ecosystems. Li et al. (2023) found that the addition of pyrimidine, oxime, azine and dimethyimidazole inhibited the abundance of nitrogen cyclis-related genes (such as *AO-AMOA*, *AOB-amoA*, *nirK*, *nirS* and *nifH*) and carbon fixation genes (such as *cbbL* and *cbbM*) in the soil, thereby affecting the nitrogen fixation and carbon storage capacity of the soil. It indirectly affects the fertility and structure of the soil. However, the current research on pesticide-contaminated soil mainly focuses on dryland crops such as corn and wheat, and the research on rice crops is relatively limited.

The longer flooding time, lower REDOX potential and poor ventilation conditions of paddy soil promoted the anaerobic metabolism among soil microorganisms. These unique environmental conditions greatly limited the activity of aerobic microorganisms, and at the same time established suitable habits for anaerobic microbial communities, forming unique microbial community structure and functional characteristics (Fan et al., 2021). Zou et al. (2024) found that the bacteria of Chloromyces and Proteobacteria have a high relative abundance in paddy field environment, and most of them are anaerobic or facultative anaerobic bacteria, which can adapt to the anoxic environment and participate in the cycle of various elements. Considering the importance of rice cultivation to the global food supply, it is of great significance to evaluate the effects of rice application on paddy microbiome for sustainable agricultural development and ecological environment protection.

Although there have been studies in recent years that have focused on the overall effects of pesticides on microbial communities in paddy fields, most of these studies have been limited to the overall community level, and less attention has been paid to subpopulations associated with ecological niches (Serbent et al., 2021). The limitation of this type of research is that it is unable to deeply reveal the functional differences and interactions of microorganisms across different ecological niches, thus limiting our understanding of microbial community functions and ecological processes under the influence of pesticides. In fact, analyzing niche subpopulations is critical because these microorganisms directly reflect the subtle differences and complexity of soil ecological functions. In theory, pesticide application may affect the cycling of carbon, nitrogen, phosphorus and other elements in soil by altering microbial community structure and functional gene expression (Yang et al., 2024). Therefore, it is urgent to study the effects of pesticides on soil microorganisms from the perspectives of community assembly, network and function of microbial subcommunities.

In addition, most studies on the evaluation methods of the impact of pesticides on microorganisms were carried out by microcosmic experiments, and were limited to one or several pesticides, which could not reflect the real impact of pesticide application on microbial communities at field scale and during the whole growth cycle (Kolekar et al., 2019). Long-term positioning experiments can effectively avoid this defect. However, most of the current long-term positioning experiments are used to evaluate the effects of fertilization mode, and few reveal the effects of pesticide application and non-application mode on microorganisms, so it is urgent to carry out. In this study, amplification of 16S rRNA and ITS sequences were performed to analyze the composition, assembly and biological networks of specialist and generalist subcommunities in pesticide-contaminated and pesticide-free soils within paddy fields. Our study has two main objectives: (1) to identify differences in the diversity and structure of specialists and generalists in soil microbiomes with and without pesticide application; and (2) to explore the models and mechanisms of biological interactions in these soils. Based on this, we hypothesize that specialist and generalist species within varied ecological contexts are likely to demonstrate significant differences in community diversity, structural composition, microbial interactions, and assembly patterns in soils with and without pesticide application.

## 2 Materials and methods

### 2.1 Soil sample collection and processing

The field experiment was located in Qianjiang (30°53′N, 111°83′E), Hubei province, China (established in 2016 and harvested in 2024). The climate in this experimental site is temperate, humid. The mean annual temperature is 15–17°C. The mean annual precipitation is 1,000 mm. The soil was at the experimental site is yellow brown soil. Rice is a cornerstone of Hubei’s agricultural sector, playing a vital role in the local economy, food security, and environmental sustainability. However, due to the frequent use of pesticides, ecological risks such as soil pollution and destruction of soil microbial communities and soil fertility have decreased. In particular, it can affect soil microbial communities, altering their diversity and function. In recent years, some studies have shown that not using pesticides reduces environmental pollution, enhances soil health, improves rice quality and reduces production costs. Therefore, to reveal the effects of both traditional and non-pesticide farming methods on microbes, this study constructed “Long-term pesticide exposure” and “non-pesticide” treatment.

Two treatments are included: HH (non-pesticide) and HP (Long-term pesticide exposure). The experiment employed a split-plot design, comprising plots measuring 10 m × 10 m, with each treatment replicated five times. The treatment is carried out in accordance with local application practices, chlorantraniliprole is typically applied at a rate of 10–20 mL of 5% suspension per acre, diluted with 30 kg of water, during the initial stage of pest infestation. Tebuconazole is typically applied at a rate of 10–15 mL of 43% suspension per acre, diluted with 30–45 liters of water for spraying. The optimal application timing includes the early tillering stage, maximum tillering stage. Irrigation, fertilization and other measures are in line with local farmers’ habits.

The sample was collected in 2024 and has 8 years of field management history. Samples were collected by 5-point sampling method, transported to the laboratory with dry ice and frozen at −80°C. The results of soil physical and chemical properties and pesticide residues showed that soil pH of 6.75, a soil organic matter (SOM) content of 22.76 g/kg^−1^, a total nitrogen (TN) content of 1.53 g/kg^−1^, a total phosphorus (TP) content of 0.88 g/kg^−1^, an available nitrogen (AN) content of 121.30 mg/kg^−1^, an available phosphorus (AP) content of 19.16 mg/kg^−1^, an available potassium (AK) content of 59.10 mg/kg^−1^ and a bulk density of 1.20 g/cm^−3^. In the HP treatment soil, the residual amounts of chlorantraniliprole and tebuconazole were 0.19 mg/kg and 0.45 mg/kg, respectively, whereas these pesticides were undetectable in the HH treatment soil.

### 2.2 High-throughput sequencing

#### 2.2.1 DNA extraction

Extract DNA from 0.25 g of fresh soil samples with the OMEGA Soil DNA Kit (D5635-02), following the manufacturer’s detailed protocols (Omega Bio Tek, Norcross, GA, United States). Use a Nanodrop ND-2000 UV Visible spectrophotometer (NanoDrop Technologies, Wilmington, DE, United States) to evaluate the quality and quantity of the extracted DNA. Perform gene amplification with the 799F (5′-AACMGGATTAGATACCCKG-3′) and 1193R (5′-ACGTCATCCCCACCTTCC-3′) primers targeting the V5–V7 region of the bacterial 16S rRNA, and the ITS1F (5′-CTTGGTCATTTAGAGGAAGTAA-3′) and ITS2R (5′-GCTGCGTTCTTCATCGATGC-3′) primers for the ITS1(b) region of the fungal ITS rRNA. The resulting amplicons were sequenced on the Illumina MiSeq platform at Personal Biotechnology (Shanghai, China). The raw reads were processed using QIIME2. After filtering, the reads were denoised and clustered into amplicon sequence variants (ASVs) using DADA2. Bacterial and fungal ASVs were assigned to taxonomic groups based on the SILVA database and the UNITE database, respectively. All sequencing data are deposited in the NCBI and can be accessed in the short read archive (SRA) under accession number PRJNA1279364 and PRJNA1279369.

### 2.3 Data analysis and statistics

We classified ASVs as specialists, generalists, or neutral taxa by comparing their niche breadth’s 95% confidence interval to null distributions (Wang et al., 2023). Niche breadth was calculated using the spaa package, and ASV occurrence frequency was determined through 1,000 random rearrangements with the EcolUtils package (Wang C. et al., 2024). Alpha-diversity indices were calculated using the vegan package in R. PERMANOVA and principal coordinate analysis (PCoA) were carried out with the vegan and ade4 packages in R software, respectively. The “VennDiagram” package (v1.6.20) was utilized to generate a Venn diagram. Functional annotations for bacterial and fungal communities were conducted using FAPROTAX and FUNGuild, respectively (Nguyen et al., 2016). Statistical significance was determined using *t*-tests and Wilcoxon rank-sum tests in SPSS software version 20. Neutral processes in microbiome assembly were quantified using a neutral model. The niche width was calculated using the weighted modified Levins index of Colwell and Futuyma (1971). Co-occurrence plots, generated with the “igraph” R package (Wu et al., 2023), were visualized in “Gephi” (Zhang et al., 2024). Spearman correlation coefficients were used to establish co-occurrence relationships, with |*r*| values greater than 0.6 and *p* < 0.05 being considered significant. Node size and connection thickness in the network represent connectivity and Spearman correlation coefficients, respectively. Network topology metrics, such as eigenvector centrality, degree, and closeness centrality, were analyzed using “Gephi,” where higher values signify central positions within the biotic network (Sun et al., 2024). Nodes were classified into four groups: module hubs (Zi > 2.5), network hubs (Zi > 2.5 and Pi > 0.62), connectors (Pi > 0.62), and peripherals (Zi < 2.5 and Pi < 0.62). Among these, network hubs, module hubs, and connectors were identified as keystone taxa (Varsadiya et al., 2021).

## 3 Results

### 3.1 Proportions of microbial generalists and specialists in pesticide-contaminated soils

**Figure 1:**
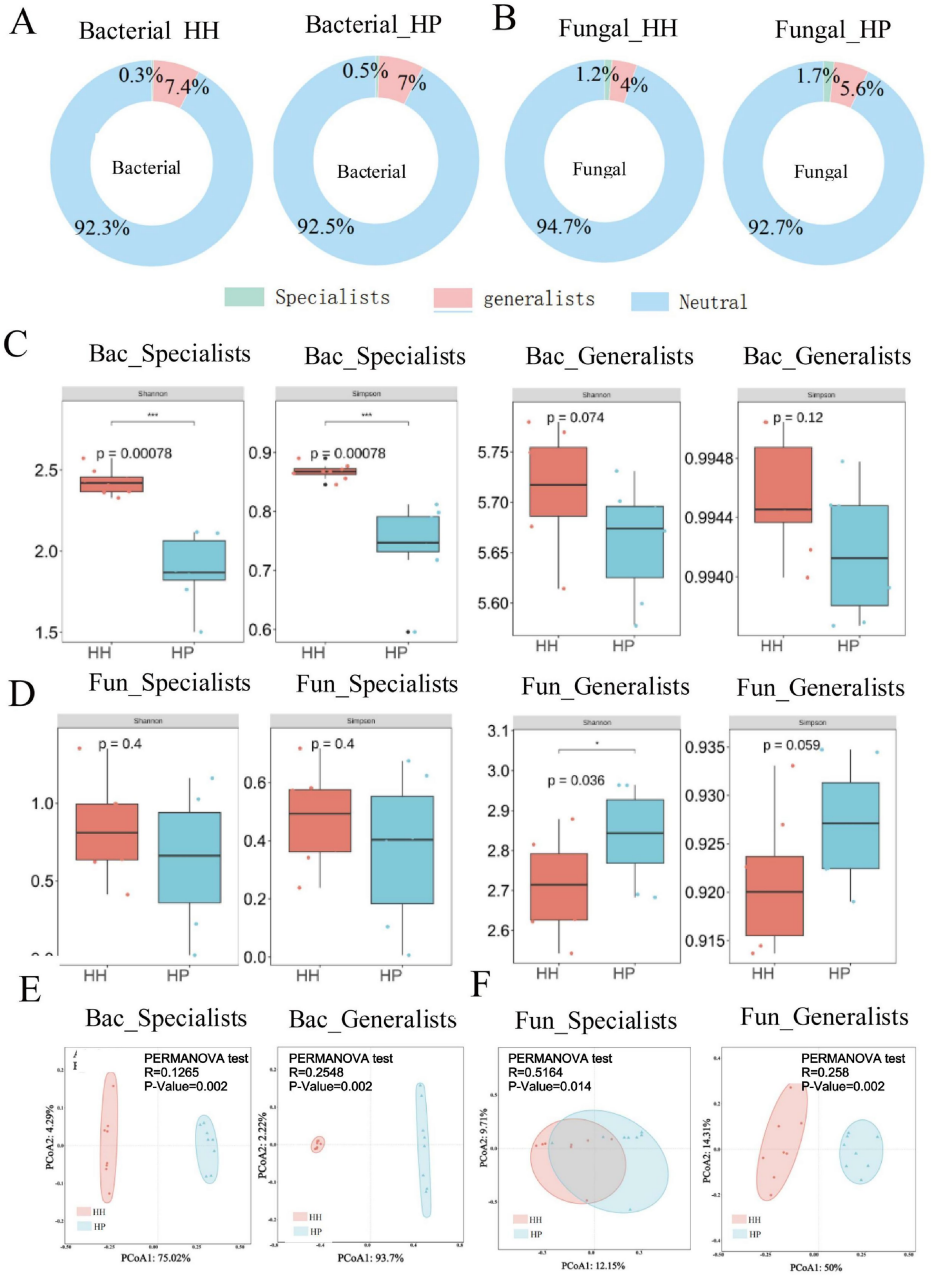
Generalist and specialist proportions in bacterial **(A)** and fungal **(B)** communities via ASVs in HH and HP. Alpha-diversity comparison for bacterial **(C)** and fungal **(D)** species. PCoA for bacterial **(E)** and fungal **(F)** species. ^**^p < 0.01 and ^*^p < 0.05; n.s., not significant.

We adopted 16S rRNA and ITS sequence analysis to examine the changes in soil bacterial and fungal communities under HH and HP treatments. According to high-throughput sequencing, a total of 142,981 bacterial reads and 54,308 fungal reads were obtained. After quality control, 41,531 ASVs for bacteria and 36,388 ASVs for fungi were acquired. ASVs from bacterial and fungal generalists and specialists made up a small fraction of the total microbiome, but their relative abundance varied significantly between the two communities. Bacterial generalists were more prevalent, comprising 7.4% in HH treatment and 7.0% in HP treatment, compared to fungal generalists, which accounted for 4.0% in HH and 5.6% in HP (Figure 1A). In contrast, fungal specialists were more abundant, making up 1.2% in HH and 1.7% in HP, while bacterial specialists accounted for only 0.3% in HH and 0.5% in HP (Figure 1B). In addition, the proportion of specialists was higher in the HP treatment than that in HH treatment. The Simpson and Shannon indices were used to evaluate bacterial (Figure 1C) and fungal (Figure 1D) diversity. The results showed that both the Simpson and Shannon indices for bacteria under HH treatment were significantly higher than those under HP treatment (*p* < 0.05). In contrast, the both indices for fungi were higher in the HP treatment than in HH. Furthermore, the generalists of both bacteria and fungi were more abundant than the specialists. PCoA and ADONIS revealed significant compositional differences in bacterial (*p*-value of 0.002 for both specialists and generalists; Figure 1E) and fungal (*p*-value of 0.014 for specialists; p-value of 0.002 for generalists; Figure 1F) communities between HH and HP treated soils (*p* < 0.05).

### 3.2 Compositional structure of microbial generalists and specialists in pesticide-contaminated soils

**Figure 2:**
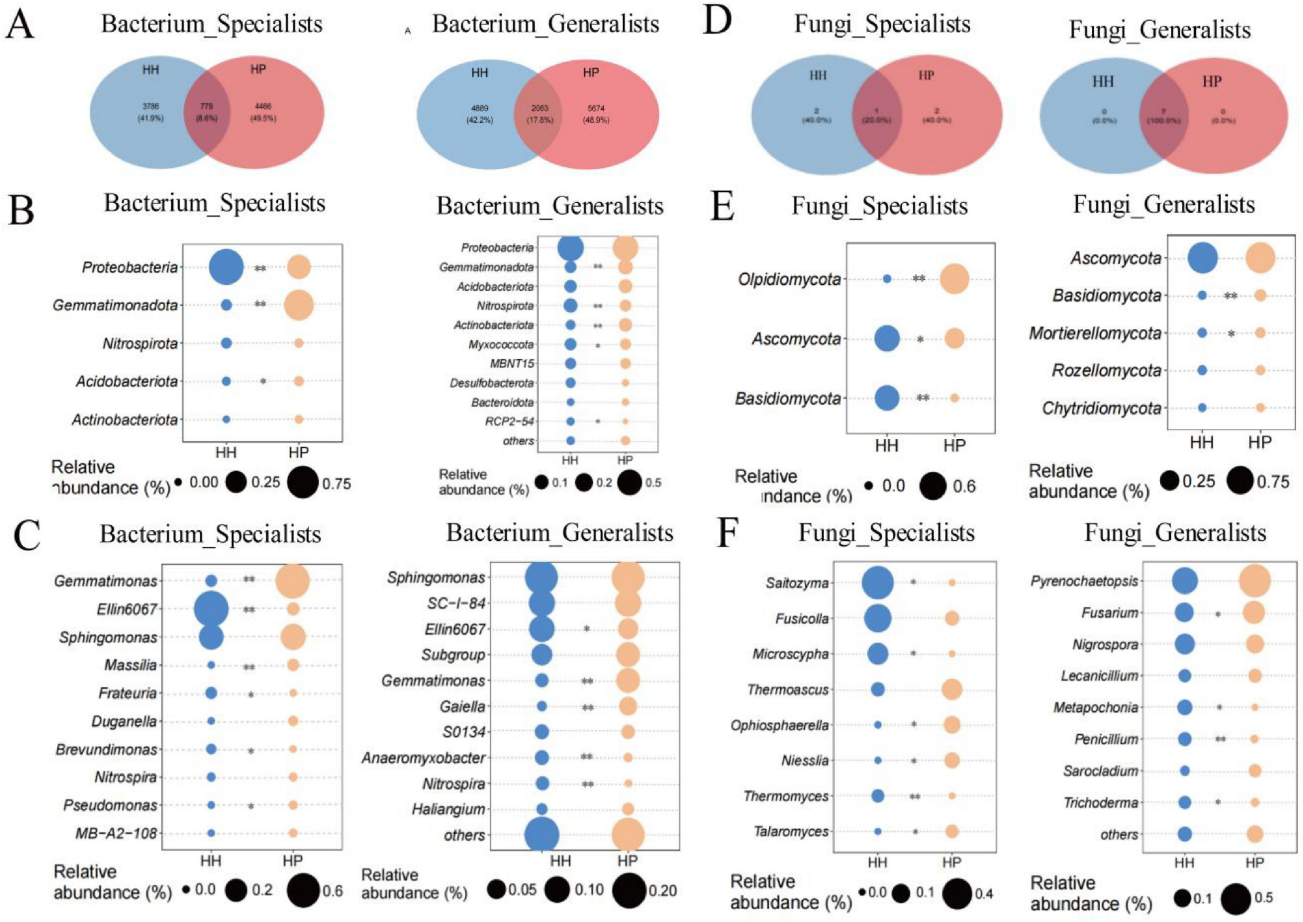
Specialist and generalist abundances in HH and HP. Venn diagram of shared bacterial taxa between plastisphere and soil **(A)**. Bacterial taxa abundances at phylum **(B)** and genus **(C)** levels. Venn diagram of shared fungal taxa **(D)**. Fungal taxa abundances at phylum **(E)** and genus **(F)** levels. ^**^p < 0.01 and ^*^p < 0.05.

Through analysis of bacterial communities, we found that 779 ASVs (8.6%) from the specialists and 2063 ASVs (17.8%) from the generalists overlapped between the HH and HP soils (Figure 2A). The bacterial specialists included five phyla, namely *Proteobacteria*, *Gemmatimonadota*, *Nitrospirota*, *Acidobacteriota*, and *Actinobacteriota* (Figure 2B). Among these, the specialist Gemmatimonadota showed significantly higher relative abundances in HH than in HP, while *Proteobacteria* and *Acidobacteriota* were also significantly increased in HH (*p* < 0.05). In the bacterial generalists, *Gemmatimonadota* and *Actinobacteriota* displayed notably higher prevalence in HP than in HH, while *Nitrospirota*, *Myxococcota*, and RCP2—54 were significantly increased in HH (*p* < 0.05) (Figure 2C). Within the 10 most prevalent genera, *Gemmatimonas*, *Massilia*, and *Pseudomonas*, classified as specialists, exhibited notably greater concentrations in HP relative to HH, while *Ellin6067*, *Frateuria*, and *Brevundimonas* were significantly increased in HH (*p* < 0.05). For the bacterial generalists, *Gemmatimonas* and *Gaiella* showed notably higher abundances in HP than in HH, while *Ellin6067*, *Anaeromyxobacter*, and *Nitrospira* were significantly increased in HH (*p* < 0.05).

In the fungal communities, one ASVs (20.0%) from the generalists and seven ASVs (100%) from the specialists overlapped between HH and HP (Figure 2D). The fungal specialists encompassed eight phyla, which were *Olpidiomycota*, *Ascomycota* and *Basidiomycota*. The fungal generalists included *Ascomycota*, *Basidiomycota*, *Mortierellomycota*, *Rozellomycota*, and *Chytridiomycota* (Figure 2E). Among these, the specialist Ascomycota was significantly enriched in HH, while the generalist *Olpidiomycota* was significantly enriched in HP. In contrast, *Ascomycota*, and *Basidiomycota* were significantly enriched in HH, and generalist *Basidiomycota*, and *Mortierellomycota* were notably abundant in the HP treatment. In the fungal specialists, the genera *Saitozyma*, *Microscypha*, and *Thermomyces* were markedly abundant in HH, while *Thermoascus*, *Niesslia*, and *Talaromyces* were abundant in HP. In the fungal generalists, *Fusarium* was notably abundant in HP, whereas *Metapochonia*, *Penicillium*, and *Trichoderma* were more prevalent in HH (Figure 2F).

### 3.3 Ecological processes driving assembly patterns of microbial generalists and specialists in pesticide-contaminated soils

**Figure 3:**
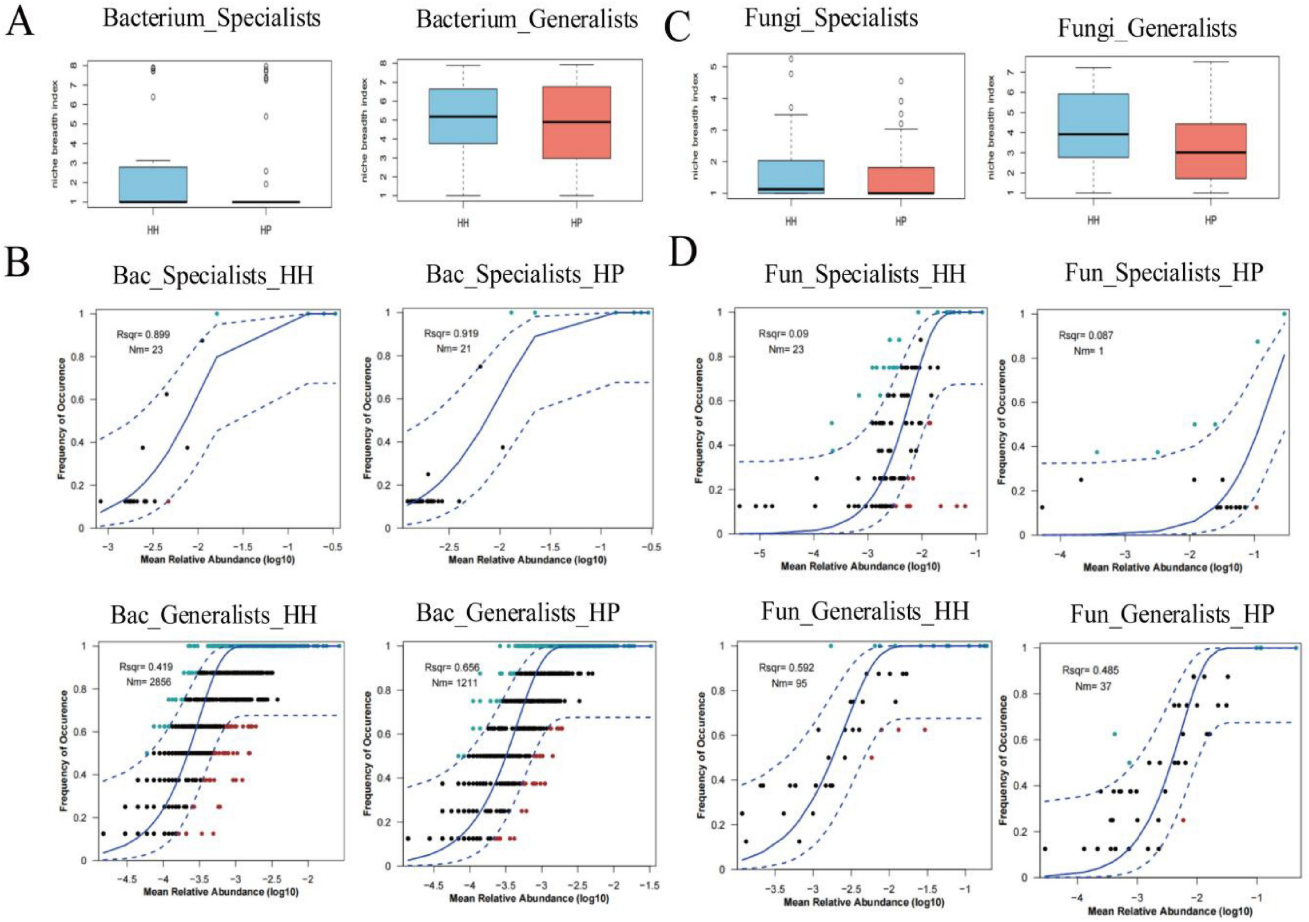
Assembly patterns of generalists and specialists in HH and HP greenhouse soils. Bacterial community niche breadth **(A)**. Neutral models for bacterial community assembly **(B)**. Fungal community niche breadth **(C)**. Neutral models for fungal community assembly **(D)**. Blue dashed lines show 95% confidence intervals, *R*^2^ indicates model fit, and Nm represents metacommunity size multiplied by immigration rate.

We analyzed the assembly patterns of both specialist and generalist species using stochastic and deterministic models, focusing on niche breadth and neutral models. The results showed that the bacterial generalists in the HH treatment had a prominently higher habitat niche breadth than that in HP, while the habitat niche breadth of specialists displayed no statistically meaningful variations between HH and HP (Figure 3A). In addition, the neutral model analysis revealed that the bacterial generalists had a higher *R*^2^ value than the specialists. In the HH treatment, bacterials demonstrated greater *R*^2^ values than those in the HP treatment (Figure 3B). Additionally, the Nm values for both generalists and specialists were significantly elevated in the HH treatment compared to the HP treatment.

### 3.4 Co-occurrence dynamics between microbial generalists and specialists in pesticide-contaminated soils

**Figure 4:**
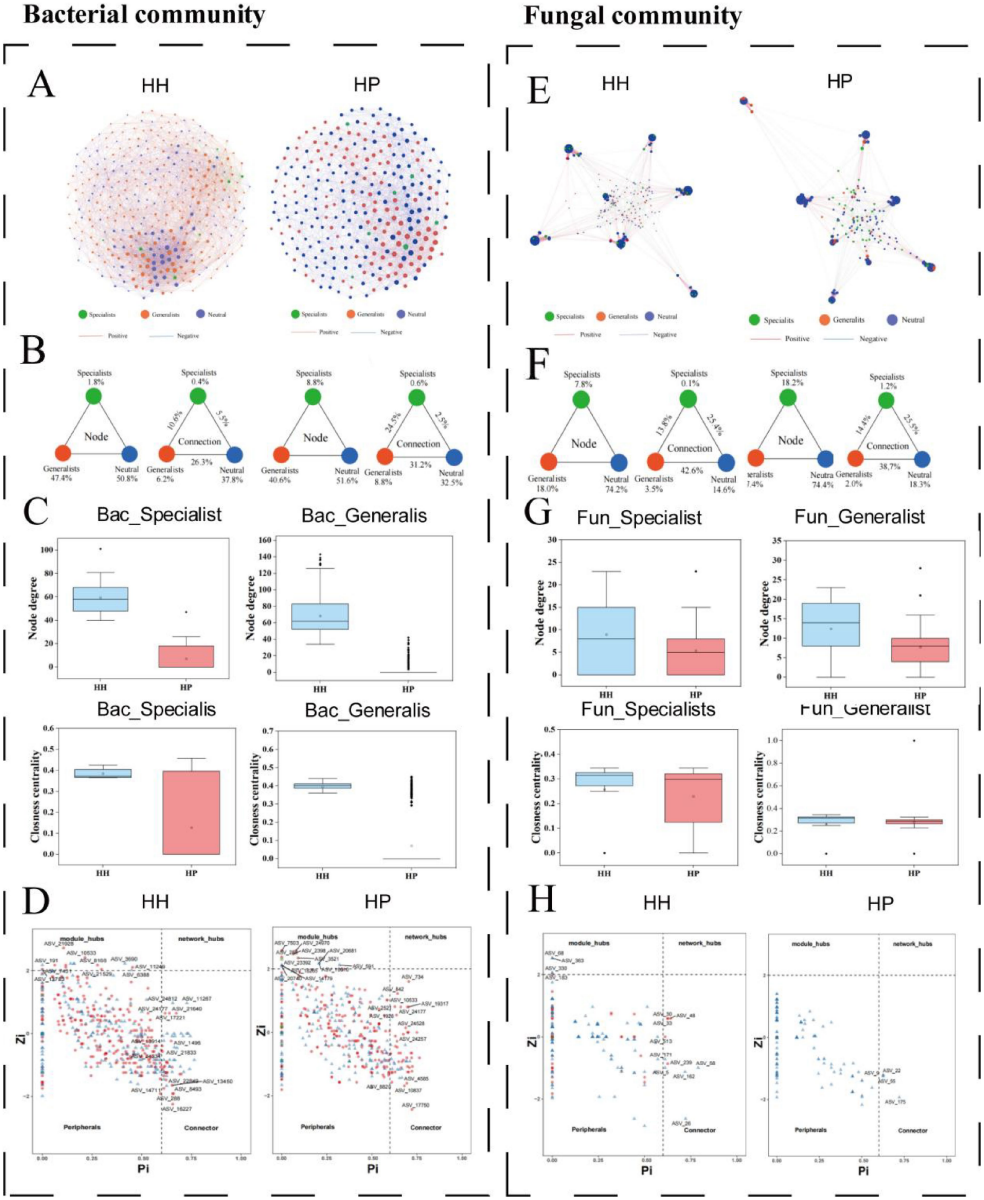
Co-occurrence patterns of specialists and generalists in pesticide-contaminated soils. Bacterial community networks **(A)**: strong correlations (*p* < 0.01). Node size reflects ASV degree, edge thickness shows Spearman’s correlation. Triangular plot of network statistics **(B)**. Bacterial community topology **(C)**. Zi-Pi plots of ASV network roles **(D)**. Fungal community networks **(E)**. Triangular plot of fungal network statistics **(F)**. Fungal community topology **(G)**. Zi-Pi plots of fungal network roles **(H)**.

In the bacterial network of the HH treatment, ASVs were classified as follows: 1.8% specialists, 47.4% generalists, and 50.8% neutral taxa (Figure 4A). The internal associations within these subcommunities comprised 0.4, 6.2, and 37.8%, respectively. External associations included 10.6% of edges connecting specialists and generalists, 5.5% linking specialists and neutral taxa, and 26.3% connecting generalists and neutral taxa. In contrast, the bacterial network of the HP treatment showed 8.8% specialists, 40.6% generalists, and 51.6% neutral taxa. The internal associations for these subcommunities were 0.6, 8.8, and 32.5%, respectively. External associations in this treatment included 24.5% of edges between specialists and generalists, 2.5% linking specialists and neutral taxa, and 31.2% connecting generalists and neutral taxa (Figure 4B). Examination of the topological characteristics across distinct subcommunities indicated that both degree and closeness centrality measures were greater for specialists and generalists in the HH treatment compared to the HP treatment (Figure 4C). The Zi-Pi plot highlighted 36 bacterial generalists and 38 neutral microorganisms as keystone species in the HH In the HP treatment, 50 generalists were identified as keystone species, and 19 neutral microorganisms were classified as keystone groups (Figure 4D). Notably, only one specialist was recognized as a keystone taxon in the HP treatment.

In the fungal community network of the HH treatment, ASVs were classified as follows: 7.8% specialists, 18% generalists, and 74.2% neutral taxa (Figure 4E). The internal associations within these subcommunities accounted for 0.1, 3.5, and 14.6%, respectively. External associations comprised 13.8% of edges linking generalist and specialist subcommunities, 25.4% connecting specialists and neutral taxa, and 42.6% connecting generalists and neutral taxa. In the fungal network of the HP treatment, the distribution of ASVs was 18.2% specialists, 7.4% generalists, and 74.4% neutral taxa. The internal associations for these subcommunities were 1.2, 2.0, and 18.3%, respectively. External associations included 14.4% of edges connecting specialists and generalists, 25.5% linking specialists and neutral taxa, and 38.7% connecting the generalist species and neutral taxa (Figure 4F). The degrees and closeness centrality values for the subcommunities of fungal specialists and generalists were comparable to those observed in the bacterial microbes (Figure 4G). The Zi-Pi plot highlighted four fungal generalists and six neutral taxa as keystone taxa in the HH treatment, while only three fungal neutral taxa were recognized as keystone species under the HP treatment (Figure 4H).

**Figure 5:**
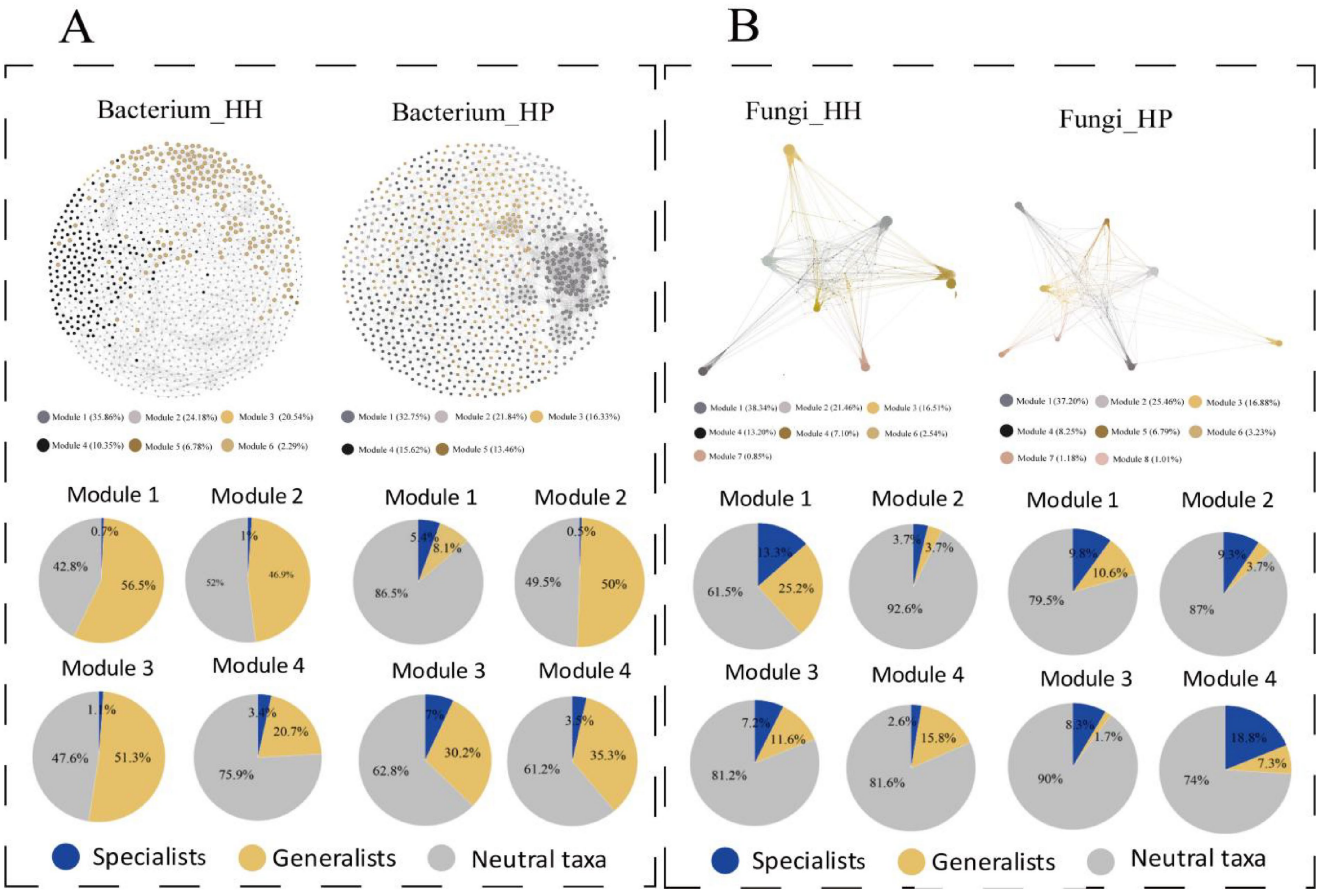
ASV co-occurrence patterns by modularity classes. Bacterial community networks in HH and HP **(A)**. Fungal community networks in HH and HP **(B)**. Top four modules are shown, nodes are color-coded by modularity classes, and pie charts display ASV subcommunity proportions per module.

Upon node modularization, the majority of nodes across all network plots were categorized into four major modules, constituting 90.93, 91.54, 89.51, and 87.79% of the total nodes in the HH of the bacterial community, HP of the bacterial community (Figure 5A), HH of the fungal community, and HP of the fungal community (Figure 5B), respectively. In each module, the abundance of specialized subcommunities in the fungal network was higher than that in the bacterial network. Furthermore, the proportion of subcommunities in each module also showed a large difference between HH and HP, suggesting they play a pivotal role in mediating stress resistance pathways across the microbial community. In the bacterial community, the HP network showed a higher abundance of generalists in each module compared to the HH network. However, specialized microorganisms occupy a higher proportion in each module in HP.

### 3.5 Potential functions of microbial generalists and specialists in pesticide-contaminated soils

**Figure 6:**
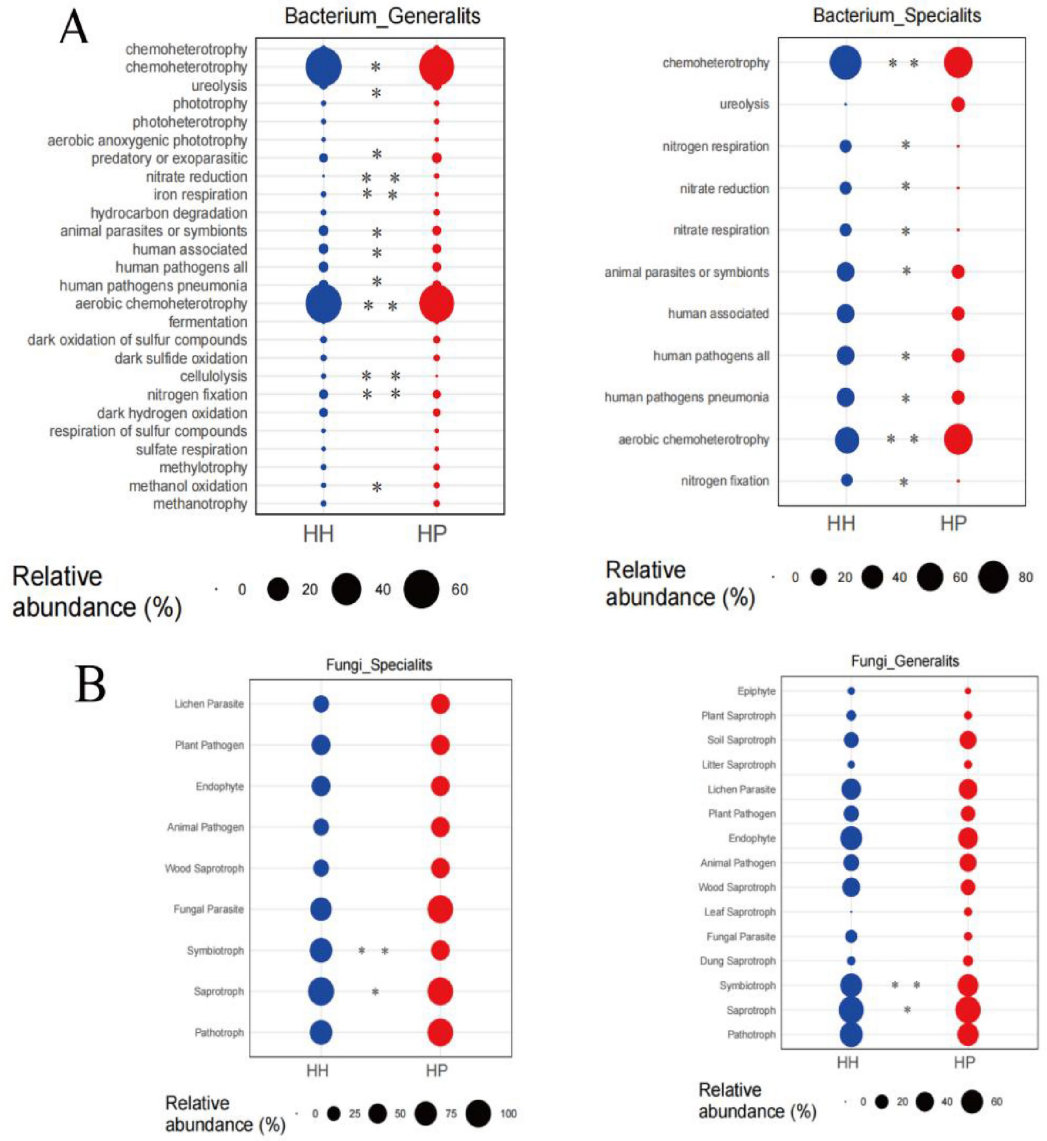
Generalist and specialist’s functions in HH and HP. Bacterial functional analysis via FAPROTAX **(A)**. Fungal functional analysis by FUNGuild **(B)**. ^**^p < 0.01 and ^*^p < 0.05; n.s., not significant.

A thorough examination regarding the functional profiles of both bacterial (Figure 6A) and fungal (Figure 6B) communities was carried out using FAPROTAX. In both treatments, a total of nine significantly differentially expressed genes were identified, associated with functions such as chemoheterotrophy, nitrogen respiration, nitrate reduction, nitrate respiration, animal parasites or symbionts, human pathogens related to pneumonia, aerobic chemoheterotrophy, and nitrogen fixation within the bacterial generalists. Notably, all these genes exhibited significantly higher expression levels in the HH treatment than in the HP treatment, except aerobic chemoheterotrophy and human pathogens. Additionally, we observed significant differences in 12 genes between HP and HH. Specifically, genes related to chemoheterotrophy, cellulolysis, and nitrogen fixation were significantly downregulated in the bacterial specialists of the HP treatment compared to the generalists of the HH treatment (*p* < 0.05). For fungi, the niche width of specialized subcommunities is lower than that of generalized subcommunities, which is consistent with bacterial communities. Pesticide pollution exerts additional environmental pressure on soil microbial communities, restricting the spread and growth of fungi. This pressure weakens the random processes of the fungal community, thereby leading to a decrease in the Nm value (Figures 3C,D). In the fungal specialists and generalists, the relative abundance of Symbiotroph and Saprotroph genes in the HP treatment was significantly lower than that in HH.

## 4 Discussion

### 4.1 Diversity of microbial specialists and generalists in pesticide-contaminated soils Life Science Identifiers

The effects of pesticides on soil microbial communities are complex and multifaceted. Research indicates that pesticide application can disrupt the normal activities of soil microorganisms, which are sensitive to environmental changes and serves as important biological indicators to evaluate soil quality. Albaseer et al. (2024) and Wang H. et al. (2024) found that microbial community changes in soils with pesticide residues were significantly affected by these residues. Specifically, pesticide residues were strongly correlated with soil microbiome characteristics, positively associated with the relative abundance of 113 bacterial and 130 fungal taxa, many of which are known to degrade pesticides. These taxa are often linked to pesticide degradation and nutrient cycling. Specialists and generalists play distinct roles in soil energy and nutrient cycling, with differing strategies for utilizing carbon and nutrient resources. At present, research on these subcommunities in pesticide-contaminated soils is limited. Specialists are generally more adaptable to specific environmental conditions and may utilize resources more efficiently in organic-rich environments, providing them with a competitive advantage. This may be the reason for the high abundance of bacterial and fungal specialists observed in pesticide-contaminated soils in this study. Chaudhry et al. (2005) noted that soil pollution can deplete sensitive microbial groups in the rhizosphere while enriching tolerant groups, suggesting that organic pollutants can negatively impact microbial diversity and alter community structure. This study found that the α-diversity of bacterial specialists and generalists was notably greater in the HH treatment compared to the HP treatment, indicating that HH provides a more abundant and varied habitat for generalists. This finding aligns with previous studies on the alterations in bacterial and fungal communities in organically polluted environments. Bacterial generalists exhibit strong adaptability to environmental changes and can thrive in various environments. In soils contaminated with organic matter, bacterial generalists can better adapt to environmental changes and utilize diverse resources for growth and reproduction. In contrast, bacterial specialists are more dependent on specific environmental conditions and are more sensitive to environmental changes, which may lead to a decrease in their diversity. Fungi possess a remarkable ability to decompose complex organic matter. In soils contaminated with organic pollutants, they can utilize their enzyme systems to break down these pollutants into simpler organic or inorganic substances, thus providing themselves with a rich source of nutrients (Mo et al., 2021; Tortella et al., 2005). This capability explains the high α-diversity of fungal generalists in the HP treatment and the low α-diversity of bacterial specialists in the HH treatment. These findings highlight the flexibility and habitat preferences of microorganisms, suggesting possible ecological niches that influence the distinct community structures of bacterial and fungal populations.

Organic pollutants are frequently utilized as C and N sources by microorganisms, providing essential nutrients that promote microbial growth and subsequently alter the microbial community structure. In this study, *Gemmatimonadota*, classified as a copiotroph, exhibited a significant increase in abundance in the HP treatment, which aligns with the results of Mujakić et al. (2023). Conversely, Zhang et al. (2025) reported that soil pollution led to a decline in sensitive rhizosphere microbial groups, such as *Proteobacteria* and *Acidobacteriota*, while enriching oligotrophic microorganisms. This suggests that the abundance of oligotrophic microorganisms is significantly reduced in soils contaminated with organic pollutants. Furthermore, pesticide pollution can indirectly affect the nitrification process by modifying the physical and chemical properties of the soil, such as pH, REDOX potential, and water content, thereby affecting the nitrifying microbial community. Organic pollution may also alter the form and availability of nitrogen in the soil (Ghaly and Ramakrishnan, 2015). For example, certain organic pollutants may absorb or complex nitrates in the soil, reducing their bioavailability and inhibiting the expression of genes involved in nitrate reduction and respiration. *Nitrospirota* is a key microorganism in the process of nitrate oxidation, capable of converting nitrite to nitrate, which is essential for the nitrogen cycle. In this study, the relative abundance of *Nitrospirota* decreased significantly in the HP treatment, consistent with the results of previous studies. Moreover, *Pseudomonas* is recognized as a highly efficient pesticide-degrading bacterium. Studies have shown that *Pseudomonas* can degrade various organophosphorus pesticides, pyrethroids, and herbicides (Kahlon, 2016). In this study, the abundance of *Pseudomonas* increased significantly in the HP treatment, likely due to its tolerance to environmental stress and its ability to degrade pollutants. Among the fungal communities, the pesticide exhibited direct toxicity to *Trichoderma*, which may account for the lower abundance of *Trichoderma* in soils treated with HP compared to those treated with HH. Due to the disruption of the balance of the soil microbial community and the decrease in the number of beneficial microorganisms, this change in community structure provides a more favorable ecological niche for the growth of pathogenic bacteria, resulting in an increase in the abundance of Fusarium in HP.

### 4.2 Effects of pesticide pollution on stochastic mechanisms of microbial communities

The composition and biogeographic patterns of microbial communities are influenced by both deterministic and stochastic mechanisms. Deterministic mechanisms, including environmental filtering that favors particular species according to their habitat needs, as well as interactions among species, encompassing antagonistic relationships such as competition and synergistic ones like mutualism, play a significant role in shaping predictable community compositions (Buscardo et al., 2024). In contrast, stochastic mechanisms contribute randomness to community assembly via elements like dispersal, genetic drift, and unforeseen environmental shifts. The randomness leads to fluctuations in community composition, allowing certain species to dominate through random occurrences. Thus, evaluating the relative roles of these mechanisms in microbial community assembly is crucial in microbial ecology (Wang Y.-C. et al., 2024). In this research, we applied neutral models to investigate the effects of stochastic and deterministic mechanisms on the formation of microbial generalist and specialist species. The findings revealed that the HP treatment notably diminished the influence of stochastic mechanisms on the assembly pattern of fungal and bacterial generalists, likely due to the decreased niche breadth of both subcommunities in the HP treatment. Conversely, bacterial and fungal specialists in the HP treatment were more likely to be influenced by deterministic mechanisms, while generalists exhibited less susceptibility to significant environmental influences. This phenomenon may result from pesticide contamination, which substantially alters the chemical and physical properties of the soil, imposing strong environmental selection pressures on the microbial community. Such pressures favor microorganisms that can tolerate or adapt to pesticide stress, while others may be suppressed or eliminated. Consequently, the structure of microbial communities is increasingly regulated by environmental factors. Moreover, the community assembly of specialists is more influenced by environmental factors, and pesticide use can enhance this deterministic mechanism. For example, changes in soil pH and pesticide concentrations directly affect the survival and propagation of specialists (Jeyaseelan et al., 2024; Li S. et al., 2023). Generalists typically possess wider ecological niches and greater adaptability, resulting in relatively high tolerance to pesticides. This adaptability allows them to exhibit greater resilience and diversity in pesticide-contaminated soils (Albaseer et al., 2024). Stochastic mechanisms highlight the importance of unpredictable occurrences, such as dispersal constraints and historical contingencies, in the assembly of both generalists and specialists, thereby contributing to network stability. In this context, these subcommunities exhibit enhanced adaptability to environmental shifts, as the unpredictability of assembly patterns cultivates varied structures capable of adjusting to diverse conditions. The prevalence of stochastic mechanisms indicates that a wide range of microbial species, including those with distinct functional attributes, may coexist due to random events rather than deterministic selection forces. This dynamic can result in heightened microbial diversity within these settings, potentially improving the functional capacities of the soil microbiome, such as nutrient cycling, organic matter breakdown, and overall soil health preservation (Yang et al., 2022). Understanding the influence of stochastic mechanisms on community assembly can shape agricultural methods. For example, practices that encourage environmental variability, like crop rotation, intercropping, and maintaining diverse plant cover, can support microbial diversity and functionality. These strategies may also reduce the negative impacts of pesticide residues on soil health by fostering resilient and functionally diverse microbial populations. Additionally, acknowledging the predominantly stochastic nature of microbial community assembly can inform bioremediation approaches. Introducing or enhancing diverse microbial communities may improve the breakdown of pesticide residues and other pollutants. Promoting stochastic assembly mechanisms can aid in establishing robust microbial populations capable of degrading contaminants under fluctuating environmental conditions. In conclusion, the effects of stochastic mechanisms on microbial community assembly are intricate and can profoundly influence ecological resilience, microbial diversity, soil management techniques, and bioremediation initiatives.

### 4.3 Different functions of microbial generalists and specialists in the biological networks of pesticide-contaminated soils

Biotic networks provide critical understanding of the connections present in microbial food webs. This study identified a significant increase in the percentage of both bacterial and fungal specialists among the network nodes in the HH treatment, indicating that specialists generally exhibit more complex connections and enhanced robustness within microbial networks. Specialists can improve their survival in polluted environments through interactions (such as symbiosis and competition) with other microorganisms. This result aligns with the research constructed by Chen et al. (2024), who emphasizes the restricted environmental adaptability of specialists, making their community structure particularly vulnerable to shifts in environmental conditions. Conversely, we noted a decrease in the percentage of bacterial generalists and their internal connections under the HP treatment. This decline may result from intensified competition among microorganisms, as limited resources are contested by a larger number of microorganisms. Additionally, the competition between bacteria and fungi may become more pronounced and in turn affects the connectivity of microbial networks (Maddela, 2024).

Node degree and closeness centrality are critical concepts in microbial network, providing insights into the connectivity, significance, and overall structural characteristics of each node (Lupatini et al., 2014). In this study, we observed that the node degree and closeness centrality in the HP treatment were lower than those in the HH treatment, indicating that both specialists and generalists exhibited reduced connectivity and centrality, resulting in a deviation from central positions in the network. These changes are closely related to a deterioration of network stability. In healthy ecosystems, higher node degree and centrality are generally associated with greater network stability and resistance. Pesticide pollution may disrupt the stability of microbial functional communities, resulting in changes in the roles of microorganisms within the ecosystem and a decline in the overall function of microbial communities (Ke et al., 2022).

Key species play a vital role in improving biotic connectivity within microbial communities, which can affect their functionality and structural composition (Philippot et al., 2021). This study found a higher concentration of keystone taxa within the bacterial and fungal networks of the HH treatment relative to the HP treatment. Additionally, the only identified keystone taxa were also found in the HP treatment. These findings suggest that disturbances caused by pesticide residues amplify the functions of both generalist and specialist species, thereby aiding in the preservation of ecological balance and operational integrity within the ecosystem.

Modularity is an important property of microbial networks to reflect the degree of niche differentiation among microbial communities. Each module may represent a specific niche or resource utilization pattern, with microorganisms within the module efficiently utilizing limited resources through synergistic interactions (Alcalá-Corona et al., 2021; Kang et al., 2022). In this study, both bacterial and fungal communities showed a higher proportion of specialists in the HP treatment compared to the HH treatment, consistent with previous findings. The high proportion of specialists in the fungal network module may be related to their role as “bridges” within the ecosystem. Studies have shown that fungi often act as “module connector” in multi-boundary community networks, which can help to maintain the integrity and stability of the network. Specialists can increase the connectivity and robustness of the network through close interactions with other organisms (Zhang et al., 2023).

### 4.4 Potential effects of microbial specialists and generalists on ecosystem function and health in pesticide-contaminated soils

Despite extensive research on the impact of pesticide pollution on microbial communities, the mechanisms affecting specialists and generalists remain incompletely understood. Given that different concentrations of pesticide residues can significantly influence microbial community function, further research is crucial. Previous studies have shown that soil organic pollution can significantly alter the structure and diversity of microbial communities, potentially inhibiting microbial respiration, nitrogen cycling, and organic decomposition. For instance, Rusănescu et al. (2024) observed that PAH contamination may suppress the nitrification and denitrification functions of microorganisms, thereby affecting the nitrogen cycling in soils. In this study, we utilized a functional prediction tool to preliminarily analyze the microbial functions in pesticide-contaminated soil, and discovered that nitrogen respiration, nitrate reduction, nitrate and nitrogen fixation in bacterial specialists were significantly decreased in the HP treatment. These gene functions and regulatory mechanisms have important implications for the nitrogen cycle. Notably, the abundance of these nitrogen cycle genes increased primarily in bacterial specialists. Generalists, which can thrive in diverse habitats, exhibit high functional plasticity. This adaptability allows generalists to extract nitrogen from the environment, improving resource utilization and enabling survival across various habitats while maintaining soil nitrogen fertility. In contrast, specialists typically have highly specific metabolic capabilities, allowing them to efficiently degrade specific complex organic matter, such as cellulose (Singavarapu et al., 2023; Weimer, 2022). Cellulose, a complex polysaccharide that is widely present in plant cell walls, requires specific enzyme systems such as cellulase for degradation. Specialists have evolved extensive gene repertoires related to cellulose degradation, and the enzymes encoded by these genes can efficiently decompose cellulose, providing a competitive advantage in the environment where cellulose serves as a carbon source (Lynd et al., 2002). This phenomenon explains the increased relative abundance of cellulolysis gene in the specialists under HP treatment. To comprehensively understand the ecological roles of specialists and generalists in pesticide-contaminated soils, we recommend implementing advantageous techniques such as metagenomics, metatranscriptomics, and macroproteomics. This is of great significance for the development of bioremediation technology for pesticide contamination. We also recognize that the amount of samples collected in this study may not adequately reflect temporal and spatial variations in microbial communities associated with pesticide contamination due to geographical differences in microflora. Future research should incorporate time-series samples across broader spatial areas to yield more comprehensive views on the mechanisms of microbial assembly. This strategy will help overcome the limitations of existing sampling strategies and enhance our understanding of the effects of pesticide residues on soil microecology. Notably, the present study did not entirely consider differences in pesticide species, which could influence the generalizability of our results. Subsequent studies could integrate these factors for a more complete understanding.

## 5 Conclusion

This study comprehensively examined the microbial communities in soils with and without pesticide residues, in order to assess their impact on the specialists and generalists within the ecosystem, as well as their effects on soil health and function. The findings revealed distinct responses of the specialists and generalists between pesticide-free soils and those contaminated with pesticide residues. Regarding microbial diversity, the generalists exhibited significantly higher levels than the specialists in both bacterial and fungal populations. In addition, the diversity of specialists exhibited varying responses to pesticide contamination; pesticide residues resulted in a decrease in bacterial community diversity, while fungal diversity remained higher due to their strong organic decomposition capabilities and adaptability to environmental conditions. Furthermore, the abundance of copiotrophs microorganisms, such as *Gemmatimonadota*, increased significantly in pesticide-contaminated soils, whereas the abundance of oligotrophic microorganisms, including *Proteobacteria* and *Acidobacteriota*, decreased significantly. This shift also contributed to the accumulation of *Fusarium*. Additionally, neutral community model was employed to explore the assembly mechanisms and community stability of specialists and generalists, revealing that bacteria and fungi in pesticide-contaminated soils were more susceptible to deterministic processes, particularly among the specialists. This is due to that specialists possess narrow ecological niches and high specificity in resource use, which limits their ability to disperse across different environments. Analysis of microbial network relationships showed that the specialists and generalists of microorganisms in pesticide-contaminated soils deviated from central positions within the network. Functional prediction results indicated an reduction in genes associated with nitrogen cycling and cellulolysis in soils with pesticide residues, while genes related to human diseases significantly increased. In conclusion, this study proves that pesticide pollution disrupts the dynamics of generalists and specialists subcommunities, impedes soil nutrient cycling, and increases the likelihood of disease development in farming soils. Additional investigation is required to understand the far-reaching influence of pesticide pollution on microbial communities and their operational capabilities. This understanding will be essential for guiding the rational use of pesticides in agriculture and enhancing soil health. For instance, the use of biopesticides and avoiding the application of pesticides during the sensitive growth stages of crops can significantly reduce the destruction of microbial communities, enabling microbial experts and generalists to support soil health and plant growth more effectively.

## Data Availability

The datasets presented in this study can be found in online repositories. The names of the repository/repositories and accession number(s) can be found in the article/supplementary material.
